# Traditional Chinese exercises for motor symptoms and mobility in patients with Parkinson's disease: a systematic review and meta-analysis

**DOI:** 10.3389/fnagi.2025.1612913

**Published:** 2025-08-19

**Authors:** Fumei Yuan, Hong Wang

**Affiliations:** Department of Martial Art, Wuhan Sports University, Wuhan, China

**Keywords:** traditional Chinese exercise, Parkinson's disease, meta-analysis, randomized controlled trial, motor symptoms

## Abstract

**Objective:**

To systematically assess the impact of TCE on the efficacy of interventions targeting motor symptoms and mobility in people with Parkinson's disease.

**Methods:**

a computerized search was performed for randomized controlled trials assessing TCE intervention for motor symptoms and mobility in Parkinson's disease patients across PubMed, Web of Science, Embase, Cochrane Library, EBSCO, China National Knowledge Infrastructure (CNKI), WanFang data, and VIP database, covering the period from the inception of the databases to January 2025.

**Results:**

fourteen publications encompassing 833 Parkinson's disease patients were incorporated into the literature. A meta-analysis indicated that TCE enhanced the UPDRS-III score (MD = −4.38, 95% CI [−5.95, −2.81]), TUGT score (MD = −2.78, 95% CI [−4.02, −1.54]), and BBS score (MD = 3.68, 95% CI [2.09, 5.27]). The effect size was compared with the Minimum Clinically Important Difference (MCID), and it was found that patients could perceive the alleviation of motor symptoms (UPDRS-III). Subgroup analyses indicated that for motor symptoms (UPDRS-III), the optimal exercise regimen was Qigong (MD = −5.54) with an exercise duration of <12 weeks (MD = −4.79), <3times/week (MD = −4.67), and each session duration ≥60 min (MD = −4.86). For functional walking ability (TUGT), the optimal exercise was Tai Chi (MD = −3.41) with an exercise duration of ≥12 weeks (MD = −3.81), exercise frequency <3times/week (MD = −3.04), and each session duration ≥60 min (MD = −3.05). For balance (BBS), the optimal exercise was also Tai Chi (MD = 5.03) with an exercise duration of ≥12 weeks (MD = 3.75), <3times/week (MD = 3.73) and session duration of ≥60 min (MD = 4.16).

**Conclusion:**

This meta-analysis indicates, the TCE intervention enhances motor symptoms and mobility in people with PD, with optimal outcomes observed from exercise frequency <3times/week, each session duration ≥60 min. The duration of the patient's disease and the intervention group type influenced the effect size (MD).

## 1 Introduction

As the global population ages, the incidence of Parkinson's disease (PD) has been rising annually, establishing it as the second most prevalent neurodegenerative disorder following Alzheimer's disease (Qi et al., 2021). Surveys indicate that the prevalence of Parkinson's disease (PD) varies between 111 and 329 per 100,000 individuals, with an annual incidence rate of 10 to 18 per 100,000. It is projected that by 2030, there will be over 8.7 million PD patients globally (Tysnes and Storstein, 2017) which imposing a significant burden on both families and society (Gustafsson et al., 2025). Parkinson's disease is characterized by the degeneration of dopaminergic neurons in the substantia nigra of the midbrain (Zhang et al., 2025), resulting in dopamine deficiency in the basal ganglia (Schapira and Jenner, 2011) and impaired mobility. The primary motor symptoms of Parkinson's disease include resting tremor, muscle rigidity, bradykinesia, and postural instability (Kalia and Lang, 2015; Jankovic, 2008). In the early stage, it mainly manifested as gait difficulties, etc. As the disease progressed, in the later stage, it mainly manifested as postural instability caused by the loss of postural reflexes (Jankovic, 2008). Regrettably, the precise etiology and pathogenesis of Parkinson's Disease (PD) remain inadequately understood, and no treatment can fully eradicate the condition—be it pharmacological or surgical (Wnag et al., 2017). Prolonged medication use may lead to patient dependency and introduce various side effects and complications (Cao Zhenyu and Wang, 2024). Consequently, patients typically engage in physical rehabilitation to mitigate complications and achieve the objective of delaying and managing symptoms.

Exercise therapy, a crucial component of physical rehabilitation, can augment striatal plasticity and elevate dopamine release through physical activity, ultimately ameliorating motor problems in patients (Feng et al., 2020). Research indicates that exercise therapy can alleviate motor symptoms and enhance physical activity to some degree for PD (Ellis et al., 2021). Investigating safe, effective, and highly patient-compliant non-pharmaceutical therapy is of considerable clinical significance. Rative to investigate safe and effective non-pharmacological therapy that ensure high patient adherence. Traditional Chinese Exercise (TCE), a time-honored practice for physical and mental conditioning, amalgamates traditional Chinese philosophy (such as Taoism and Confucianism), theories of Chinese medicine (including meridian theory, qi and blood theory, and internal organ theory), and pragmatic health care. It underscores the integration of body and mind, the synthesis of stillness and movement, and the coordination of inhalation and exhalation (Sun, 2014). The movement form is uncomplicated and unrestricted by temporal and spatial constraints, facilitating learning and sustained practice among the older individuals (Li-Ling, 2013). This study concentrates on Tai Chi and the four core qigong established and validated by the State General Administration of Sport of China: Yijinjing, Wuqinxi Liuzijue, and Baduanjin. The various exercises possess distinct movement patterns and philosophical underpinnings (Supplementary material 1). Consequently, these exercises have demonstrated potential for enhancing motor function and balance (Lee et al., 2022; Yuen et al., 2021; Maciaszek and Osiński, 2010), particularly in patients with Parkinson's disease (Li G. et al., 2022; Deuel and Seeberger, 2020). It is important to acknowledge that while both Tai Chi and Qigong are categorized under TCE, each has its distinct emphasis. Tai Chi focuses on gradual, fluid movement transitions and weight shifting between legs, it has the potential to enhance the patient's motor symptoms (Li G. et al., 2024). Qigong emphasizes meditation (Gouw et al., 2019) and slow, deep, prolonged breathing, when executed in a half-squat position, it can augment lower limb strength, boost the patient's gait (Liu et al., 2016), and diminish the frequency of falls.

Current research indicates that TCE enhances patients' balance, hence decreasing the likelihood of falls (Zhong et al., 2020; Xiao et al., 2016). Clinical investigations have demonstrated that TCE ameliorates patients' motor symptoms, particularly gait and balance, by increasing brain network functionality and diminishing inflammation (Li G. et al., 2022). A meta-analysis indicated that Wu et al. (2022). TCE enhances motor symptoms, balance, and gait function in people with Parkinson's disease. Despite numerous studies on TCE interventions addressing motor symptoms and mobility in Parkinson's disease patients, notable discrepancies and deficiencies persist: (1) the absence of an optimal exercise dosage for motor symptoms and mobility; and (2) the lack of an association analysis with the minimal clinically significant change, which is crucial for patients.

This study aimed to examine the best exercise dosage (exercise type, exercise duration, exercise frequency, and session duration) for TCE intervention in patients with PD by subgroup analysis and meta-regression analysis. The analysis examines the variables influencing the intervention's efficacy (disease duration, type of control group) and compares the effect size of the TCE intervention with the Minimum Clinically Important Difference (MCID) to facilitate a more accurate evaluation of its clinical value, thereby offering a robust foundation for the formulation of clinically suitable exercise interventions for patients with PD.

## 2 Methods

### 2.1 Study protocol and registration

This systematic review followed the recommendations of the Preferred Reporting Items for Systematic Reviews and Meta-Analyses (PRISMA) and the Cochrane Handbook for Systematic Reviews of Interventions. The protocol was registered in the International Prospective Register of Systematic Reviews (CRD: 420251030888).

### 2.2 Search strategy

The search was primarily undertaken using computers, supplemented by manual efforts. The English databases examined comprised PubMed, Web of Science, Embase, Cochrane Library, and EBSCO, while the Chinese databases included CNKI, WanFang data, and VIP database. The search timeframe extended from the inception of each database to January 15, 2025. The search approach employed a blend of subject phrases and free terms to locate published literature in the databases, with citation tracing and pertinent systematic evaluations of the included material to guarantee that research were not overlooked in the electronic search. Medical Subject Headings (MeSH) terminology and keywords employed in the search strategy included: “Taichi” or “Qigong” or “Yijinjing” or “Baduanjin” or “Wuqinxi,” or “Liuzijue” and “Parkinson Disease” or “Parkinson” or “parkinsonism”. Chinese databases utilize simplified Chinese characters: “太极 (Tai Chi)” or “太极拳 (Tai Chi Chuan)” or “气功 (Qigong)” or “易筋经 (Yijinjing)” or “八段锦 (Baduanjin)” or “五禽戏 (Wuqinxi)” or “六字诀 (Liuzijue)” and “帕金森 (Parkinson)” or “帕金森病 (Parkinson Disease)” or “帕金森氏病 (Parkinson Disease)” (Supplementary material 2).

### 2.3 Eligibility criteria

The research will encompass studies that fulfill the following criteria: (1) The studies included are randomized controlled trials; (2) The participants are adult patients diagnosed with idiopathic or primary Parkinson's disease; (3) The intervention measures consist of Tai Chi and four sets of Qigong: Yijinjing, Baduanjin, Wuqinxi and Liuzijue, which may be utilized independently or in conjunction with conventional treatment or other therapies; (4) The control group and the experimental group receive identical conventional treatment, alternative treatment, no intervention, or other active interventions. The intervention type (active vs. passive) for the control group will be examined in subgroup analysis; (5) The primary outcome indicators consist of motor symptoms and functional capacity; (6)Studies composed in English.

The exclusion criteria were as follows: (1) Literature including meta-analyses, systematic reviews, conferences, and non-clinical trials was excluded; (2) Literature with repeated or duplicated publications and incomplete information was excluded; (3) Literature lacking data or from which data on the required indicators could not be extracted in the full text was excluded; (4) Literature where the intervention was a non-TCE was excluded; (5) Literature involving subjects with additional major diseases alongside PD (e.g., hypertension, diabetes, etc.) was excluded.

### 2.4 Data extraction and quality assessment

Literature obtained from multiple databases was imported into EndNote software for verification of duplicates, and after removing duplicates, two researchers (F-M Y and YW) separately and independently reviewed the titles and abstracts to exclude literature that clearly did not fulfill the inclusion criteria, including systematic reviews, conference proceedings, and non-clinical trials. Literature that satisfied the criteria was subsequently incorporated through meticulous examination of the whole text and evaluation of the outcome metrics. Upon completion of the screening, the two researchers (F-M Y and YW) conducted mutual verification, and a third individual (HW) was consulted in the event of any discrepancies in opinion. The extraction of literature data encompassed fundamental details including the first author, title, year of publication, country, as well as intervention measures, outcome indicators, and statistics including sample size, mean, and standard deviation post-intervention.

Two researchers (F-M Y and YW) employed the Cochrane Literature Risk of Bias Assessment Tool (Cumpston et al., 2019) to assess the literature across seven primary domains: (1) random allocation method; (2) concealment of allocation scheme; (3) blinding of participants and researchers; (4) blinding of outcome assessments; (5) completeness of outcome data; (6) selective reporting of study findings; and (7) additional sources of bias. Each evaluation criterion was established utilizing the three catagories of “high risk,” “low risk,” and “unclear.” Studies that fully satisfied the aforementioned quality rating criteria were assigned a grade of A; those that partially satisfied the criteria received a grade of B; and studies that did not meet the requirements at all were assigned a grade of C. In the event of a disagreement, we could request that the researchers furnish us with information. In the event of a disagreement during the review process, the opinion of a third party may be solicited.

### 2.5 Statistical analysis

A meta-analysis was conducted using Review Manager 5.4 software from the Cochrane Collaboration Network and Stata 18.0 software. The significance level for the meta-analysis was set at α = 0.05, with differences deemed statistically significant at *P* < 0.05. Continuous variable outcome indicators were represented using weighted mean difference (MD) and 95% confidence intervals (CI) to convey effect sizes. The extent of inter-study heterogeneity was evaluated using the Cochran *Q*-test (*P* ≤ 0.1 indicating significant heterogeneity) and the *I*^2^ statistic. Combined effect sizes were computed employing a fixed-effects model when I^2^ < 50% and *Q*-test *P* > 0.1, and a random-effects model when *I*^2^ ≥ 50% or *P* ≤ 0.1 (Dersimonian-Laird method). Publication bias was initially evaluated through a visual evaluation of funnel plot symmetry and subsequently quantitatively analyzed using Egger's linear regression approach, with a *p*-value of less than 0.05 indicating the presence of publication bias. High-impact studies were determined using the item-by-item exclusion method: the aggregated effect sizes were recalibrated following the sequential exclusion of individual studies, and if the revised effect size surpassed the original 95% confidence interval range, the study was deemed highly influential. All research variables (disease duration, exercise duration, exercise frequency, session duration, exercise type, and type of control group) were examined as continuous variables in subgroup analyses. The exercise dosage of the TCE intervention (exercise duration, exercise frequency, session duration) was examined as a continuous variable through Meta regression analysis (restricted maximum likelihood method). The variable values were directly obtained from the original study. Ultimately, the robustness of the findings was evaluated via sensitivity analyses, which involved modifications to the combined model (fixed/random-effects conversion) and the computation of robust ranges for the 95% confidence intervals of the effect sizes.

## 3 Results

### 3.1 Search results

**Figure 1:**
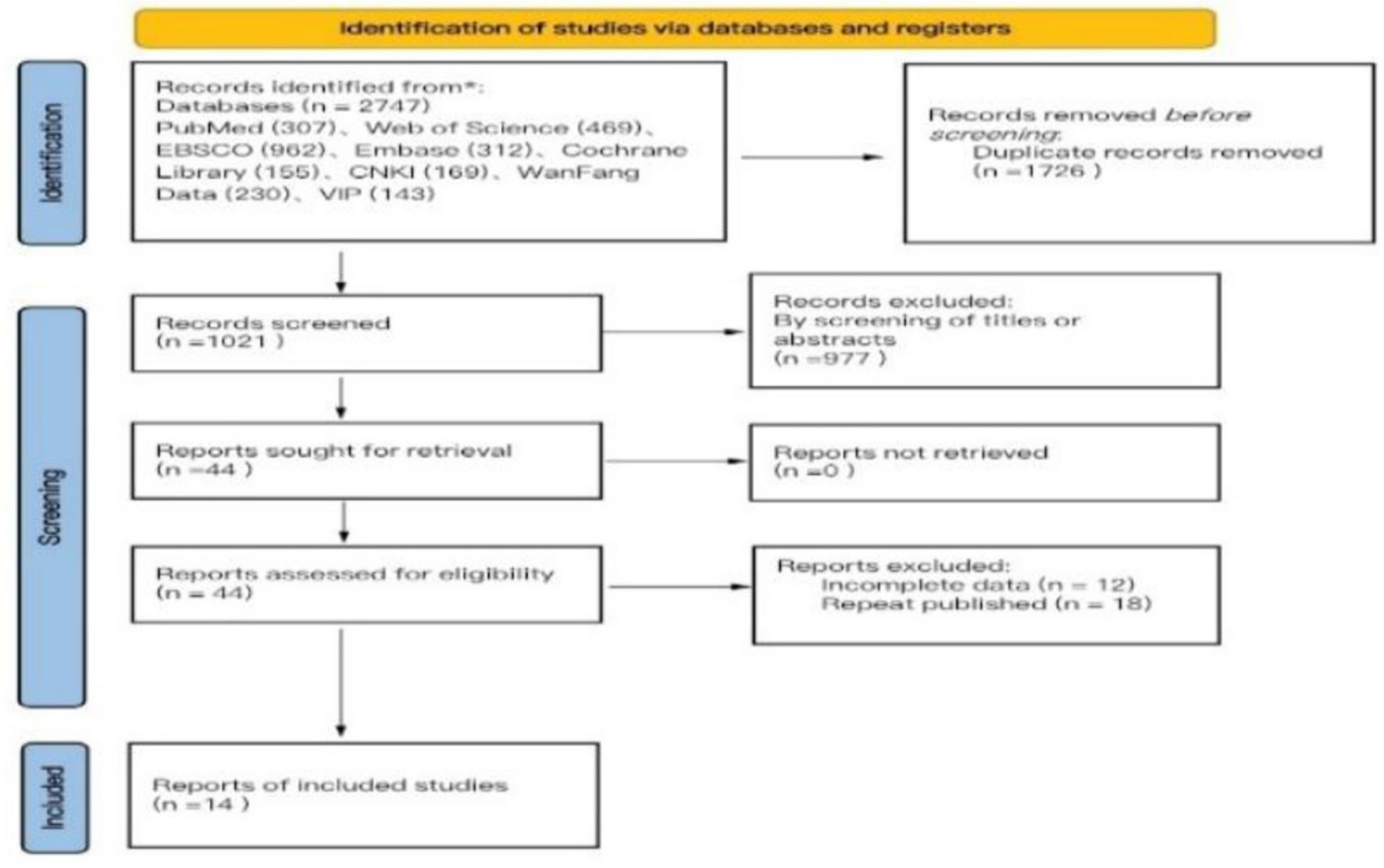
PRISMA flow diagram of study selection.

**Figure 2:**
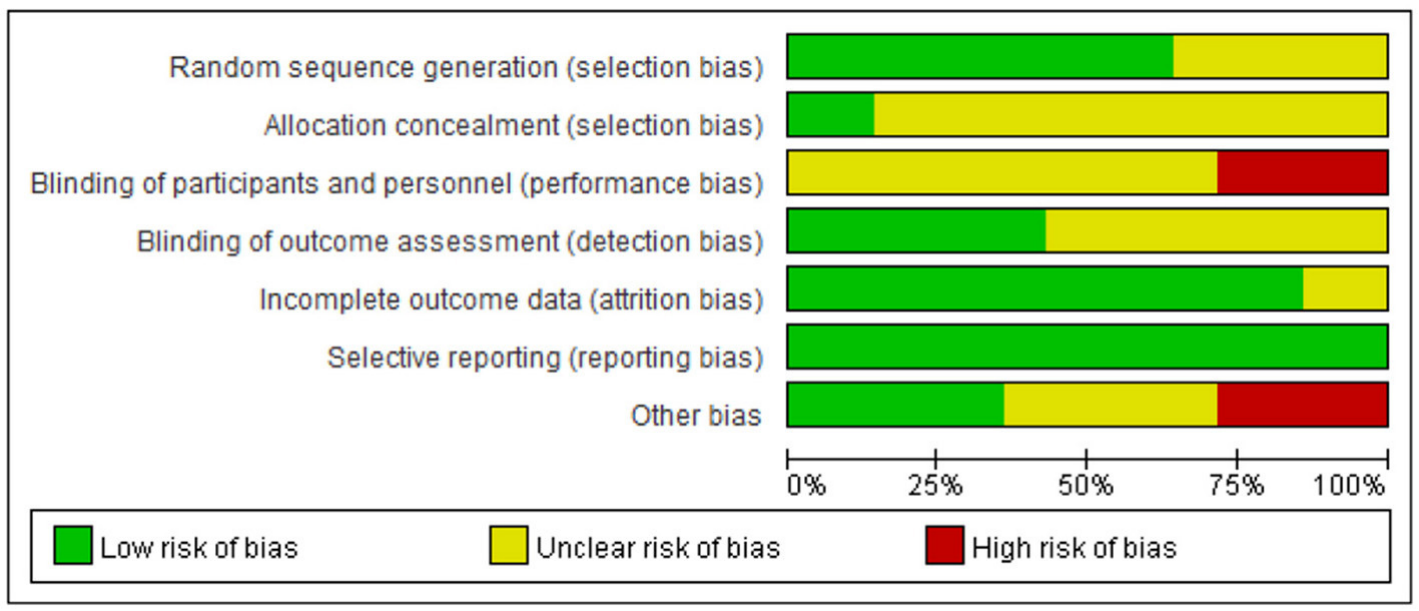
Assessment of methodological quality for RCTs.

The search methodology of this study adhered to the PRISMA guidelines for literature search and selection. A total of 2,747 articles were retrieved, comprising 542 articles in Chinese and 2,205 articles in English, which include 169 articles from CNKI, 230 articles from WanFang Database, 143 articles from VIP Database, 307 articles from PubMed, 469 articles from Web of Science, 962 articles from EBSCO, 312 articles from Embase, and 155 articles from Cochrane Library. After importing all literature into EndNote reference management software, two researchers (F-M Y and YW) conducted a systematic screening, resulting in 1,021 articles post-duplicate removal. Following the evaluation of titles and abstracts, 44 articles were identified as meeting the inclusion and exclusion criteria. Ultimately, 14 articles were selected for the meta-analysis after a thorough review of the full texts (Liu et al., 2017; Chen and Qin, 2024; You Ye, 2020; Zhu and Yi Li, 2011; Ding et al., 2021; Cao et al., 2021; Kong Xiangzhen and Hu, 2022; Li Z. et al., 2022; Li G. et al., 2022; Chang et al., 2024; Li K.-F. et al., 2024; Li et al., 2021; Gao et al., 2014; Li et al., 2012; Vergara-Diaz et al., 2018), comprising 7 articles in Chinese (Liu et al., 2017; Chen and Qin, 2024; You Ye, 2020; Zhu and Yi Li, 2011; Ding et al., 2021; Cao et al., 2021; Kong Xiangzhen and Hu, 2022) and 7 articles in English (Li Z. et al., 2022; Li-Ling, 2013; Li et al., 2021, 2012; Chang et al., 2024; Li K.-F. et al., 2024; Gao et al., 2014; Wayne et al., 2017). The literature search process is illustrated in Figure 1 (PRISMA flowchart). Among the 14 randomized controlled trials (RCTs) included, four employed permutation block randomization for group allocation, five utilized the random number table method, two concealed the allocation scheme, seven implemented blinding of the outcome assessor, eight experienced subject dropout, one encountered data loss due to subject dropout, five studies conducted follow-ups, and all included literature provided comprehensive data on primary and secondary outcome indicators (Figure 2).

### 3.2 Study characteristics

Fourteen papers were chosen, comprising a total sample size of 833 cases, with 419 instances in the experimental group and 414 cases in the control group. The fundamental characteristics of the included research are presented in Table 1.

**Table 1:** Characteristics of randomized controlled trials included in the meta-analysis.

**Author**	**Country**	**Sample size**		**Age**		**Duration of disease**		**Intervention measures**		**Exercise dosage**	**Outcome indicators**	**Hoehn- Yahr**
		**Experimental group**	**Control group**	**Experimental group**	**Control group**	**Experimental group**	**Control group**	**Experimental group**	**Control group**			
(Liu et al. 2017)	China	23	18	57.2 ± 8.0	57.1 ± 5.7	4.1 ± 1.0	4.1 ± 1.0	Qigong + basic drug treatment	Basic drug therapy	A total of 10 weeks, 5 times a week, 60min	UPDRS-III,TUGT	1–3
(Chen and Qin 2024)	China	30	30	56.10 ± 7.76	55.21 ± 6.85	2.28 ± 0.90	2.10 ± 0.86	Wuqinxi+ Jieyu Qingxin soup	Conventional treatment	Once a day for 60 min for 4 weeks	UPDRS-III,BBS	___
(You Ye 2020)	China	35	35	68.81 ± 5.02	68.49 ± 5.27	4.21 ± 0.24	4.17 ± 0.35	TaiChi balance exercise group therapy	Normal stretch + gait	A total of 24 weeks, twice a week, 60min	BBS,UPDRS-III	3
(Zhu and Yi Li 2011)	China	19	19	63.35 ± 8.72	64.83 ± 9.29	2.72 ± 1.95	2.78 ± 2.29	Taiji quan	walk	A total of 4 weeks, 10 times a week, 30–45min	UPDRS-III,BBS	1–2
(Ding et al. 2021)	China	40	40	64.1 ± 8.8	66.9 ± 8.5	11.0 ± 5.4	10.3 ± 5.2	Medicine + Tai Chi cloud hand+Fumigation	Medication + usual care	A total of 8 weeks, 5 times a week, 60min	BBS,TUGT	___
(Cao et al. 2021)	China	31	31	62.45 ± 2.87	62.97 ± 3.27	6.10 ± 0.87	5.71 ± 1.10	Wuqinxi + medicine + routine rehabilitation	Medication + regular rehabilitation training	A total of 8 weeks, 5 times a week, 30 min	BBS,TUGT	3
(Kong Xiangzhen and Hu 2022)	China	46	46	66.10 ± 5.37	65.79 ± 4.12	1.55 ± 0.41	1.52 ± 0.34	Wuqinxi+ medicine + low load exercise	Medication + low-load exercise training	Once a day for 60 min for 4 weeks	BBS	1–3
Li G. et al. (2022)	China	19	19	67.57 ± 3.95	70 ± 5.59	6.83 ± 4.09	7.76 ± 4.55	Wuqinxi	Stretching exercise	A total of 12 weeks, twice a week, 90min	MDS-UPDRS-III,TUGT	1–3
(Chang et al. 2024)	China	16	13	66.31 ± 6.54	63.15 ± 7.95	6.75 ± 5.49	6.77 ± 6.84	Taiji quan	Daily physical activity	A total of 12 weeks, twice a week, 60min	UPDRS-III	1–2
Li K.-F. et al. (2024)	China	27	27	65.59 ± 9.16	60.48 ± 11.52	4.59 ± 3.70	7.56 ± 7.95	Baduanjin	Regular exercise	For 4 weeks, 5 times a week, 40 min	MDS-UPDRS-III	1–4
(Li et al. 2021)	China	15	16	65.87 ± 6.13	63.25 ± 6.70	5.60 ± 1.72	6.13 ± 1.96	Qigong	nonintervention	A total of 12 weeks, 5 times a week, 60min	UPDRS-III,TUGT	1–3
(Gao et al. 2014)	China	37	39	69.54 ± 7.32	68.28 ± 8.53	9.15 ± 8.58	8.37 ± 8.24	Taiji quan	nonintervention	A total of 12 weeks, 3 times a week, 60min	UPDRS-III,BBS,TUGT	___
(Li et al. 2012)	USA	65	65	68 ± 9	69 ± 9	8 ± 9	6 ± 5	Taiji quan	Stretch training	A total of 24 weeks, twice a week, 60min	UPDRS-III,TUGT	1–4
(Wayne et al. 2017)	USA	16	16	65.7 ± 3.86	62 ± 7.77	2.9 ± 2.38	2.9 ± 2.20	Tai Chi training + regular health care	Routine health care	For 6 months, twice a week for 60 min	UPDRS-III,TUGT	1–2.5

Berg Balance Scale (BBS); Timed ‘Up and Go' Test (TUGT); Part III of the Unified Parkinson's Disease Rating Scale (UPDRS-III); The Movement Disorders Association revised the Uniform Parkinson's Disease Rating Scale Part III, (MDS-UPDRS-III).

### 3.3 Study quality

**Figure 3:**
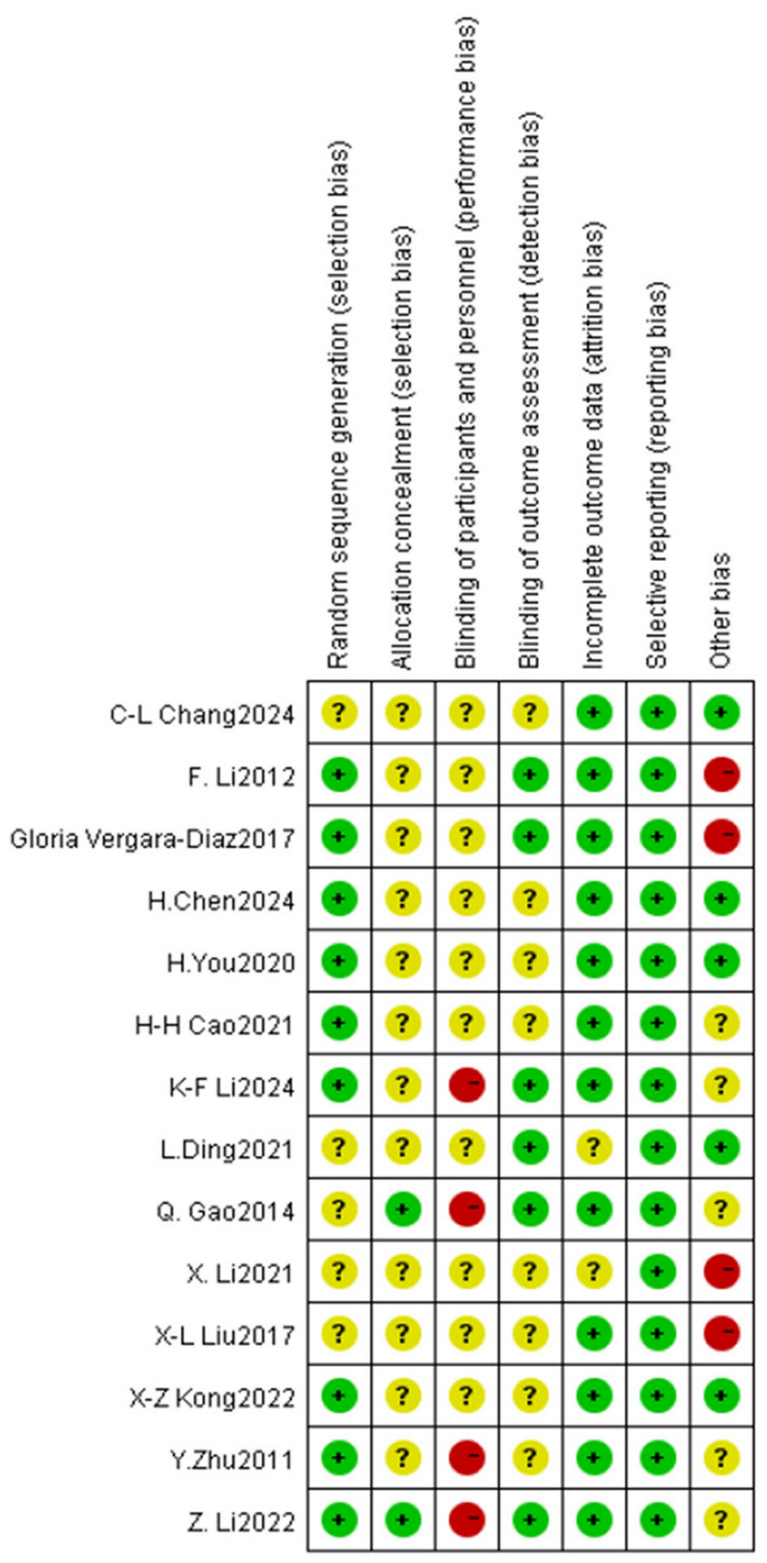
Assessment of risk of bias for RCTs.

**Figure 4:**
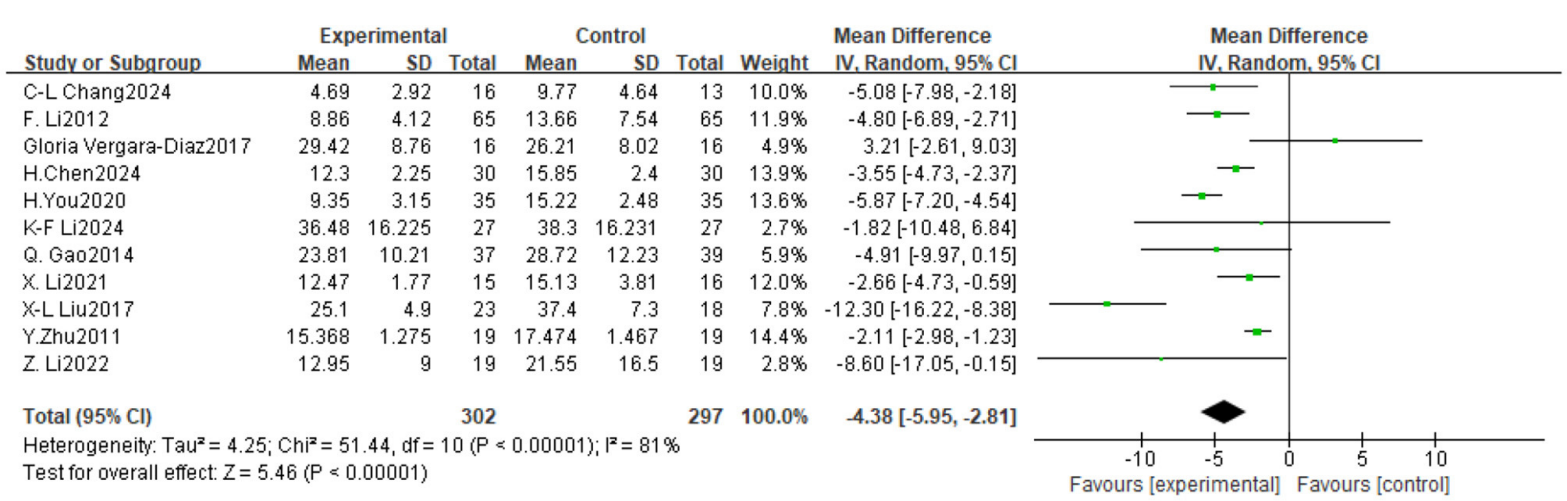
Forest plot of the effect of traditional Chinese exercise on UPDRS-III indicators of the two groups.

The methodological quality of the literature incorporated in this study was grade B, there exists a potential risk of bias, necessitating cautious interpretation of the results. The outcomes of the risk of bias assessment for the included literature are presented in Figures 3, 4 (Supplementary material 3).

### 3.4 Meta-analysis

#### 3.4.1 UPDRS-III

**Figure 5:**
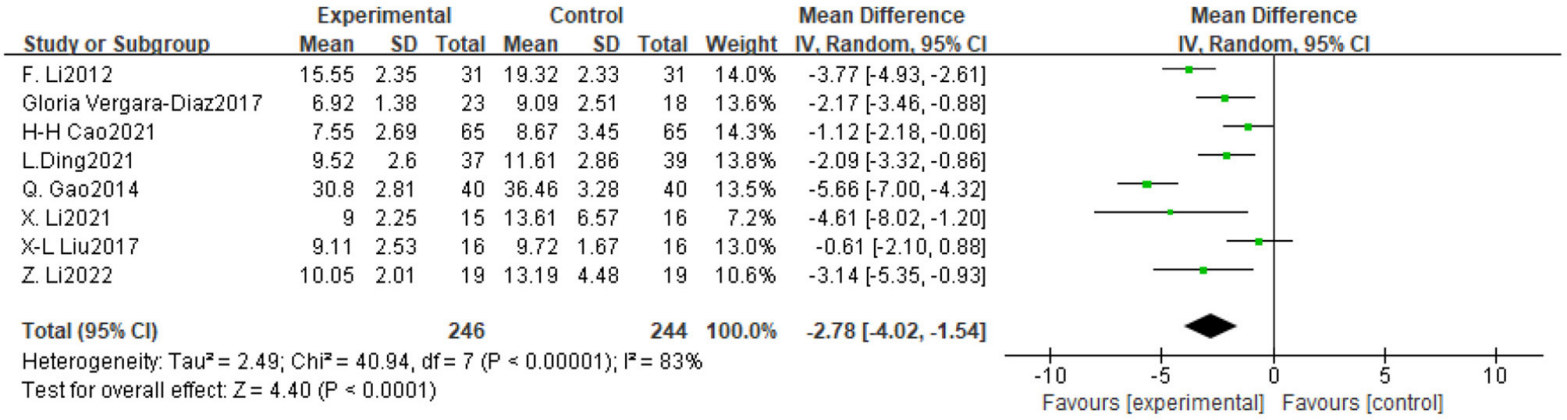
Forest plot of the effect of traditional Chinese exercise on TUGT indicators in two groups.

Of the 14 trials considered, 11 assessed the impact of TCE on motor function in patients with Parkinson's disease, involving a total of 599 participants. The findings indicated significant inter-study heterogeneity (*P* < 0.00001, *I*^2^ = 81%), hence a meta-analysis was conducted utilizing a random effects model. The findings indicated that TCE significantly enhanced motor performance in PD patients Mean Difference (MD) = −4.38, 95% CI [−5.95, −2.81], *P* < 0.00001). As illustrated in Figure 5.

#### 3.4.2 TUGT

**Figure 6:**
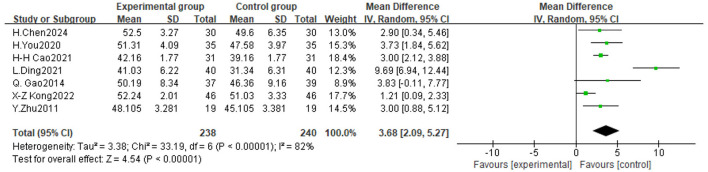
Forest plot of the effect of traditional Chinese exercise on BBS indicators of the two groups.

Eight trials assessed the impact of TCE on balance and ambulation in people with Parkinson's disease, involving a total of 490 participants. The findings indicated significant inter-study heterogeneity (*P* < 0.00001, *I*^2^ = 83%), necessitating the application of a random-effects model for the meta-analysis. The findings indicated that TCE enhanced balance and ambulation in patients with Parkinson's disease (MD = −2.78, 95% CI [−4.02, −1.54], *P* < 0.0001). As illustrated in Figure 6.

#### 3.4.3 BBS

**Figure 7:**
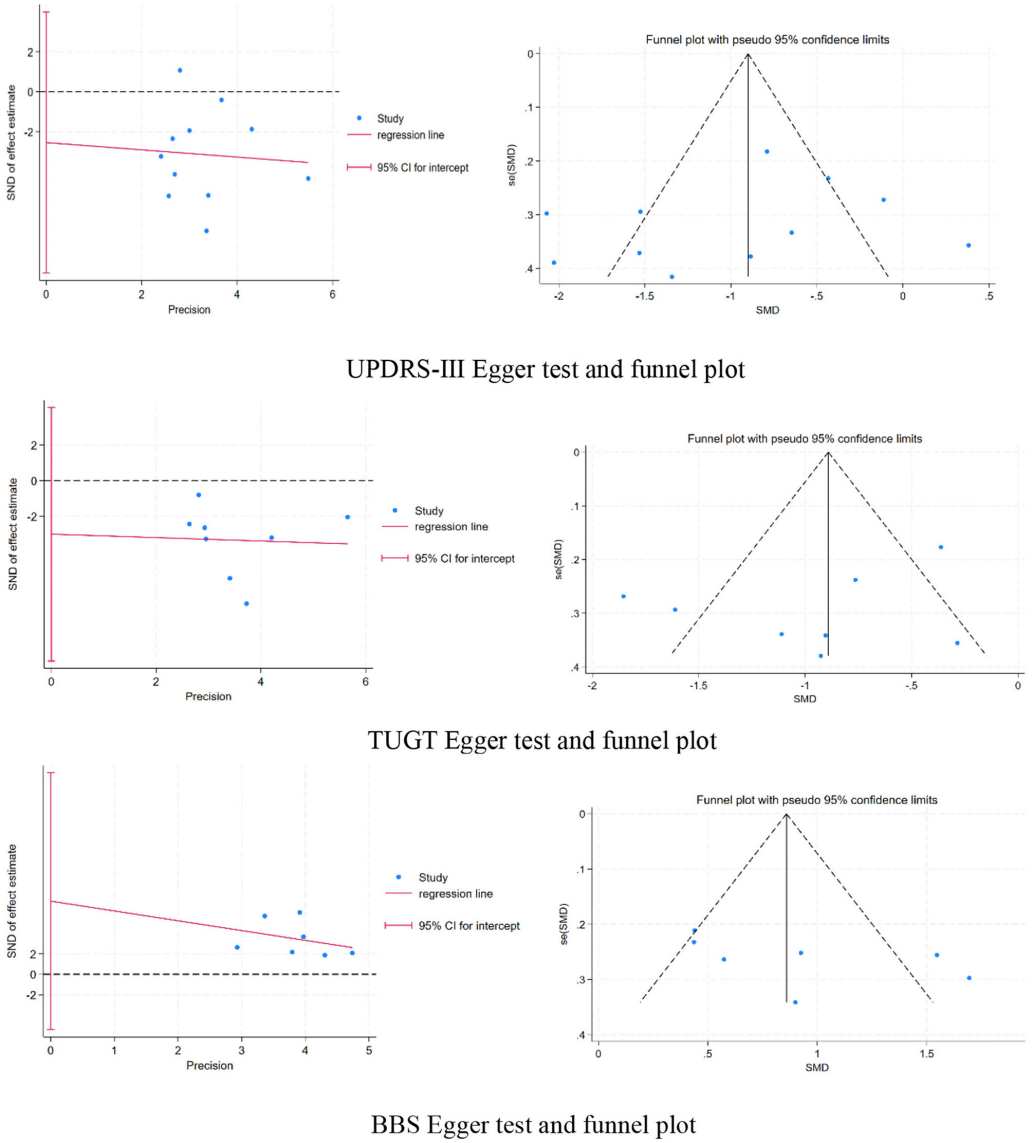
Publication bias in the included studies.

Seven trials assessed the impact of TCE on balance function in people with Parkinson's disease, involving a total of 478 participants. The findings indicated significant heterogeneity among the investigations (*P* < 0.00001, *I*^2^ = 82%), prompting the execution of a meta-analysis utilizing a random-effects model. The findings indicated that TCE enhanced balance function in patients with Parkinson's disease (MD = 3.68, 95% CI [2.09, 5.27], *P* < 0.0001). As illustrated in Figure 7.

### 3.5 Subgroup analysis of exercise dosage

#### 3.5.1 Exercise duration

The International PD Movement Disorders Society (MDS) asserts that (Obeso et al., 2017) exercise interventions must be sustained for 8–12 weeks to yield clinical benefits (Supplementary material 4). Consequently, in this study, the included experiments with exercise durations (4 weeks, 8 weeks, 12 weeks, 24 weeks, and 6 months) were categorized into two subgroups (≥12 weeks, < 12 weeks). The subgroup analyses indicated that exercise durations of ≥12 weeks and < 12 weeks significantly influenced the enhancement of UPDRS-III scores in Parkinson's disease patients (MD = −4.28, 95% CI [−5.99, −2.58] and −4.79, 95% CI [−7.66, −1.93], respectively), as well as TUGT scores, with MD = −3.81 [−5.19, −2.43] and −1.32 [−2.12, −0.52], respectively; and BBS scores, with MD = 3.75, 95% CI [2.05, 5.45] and 3.71, 95% CI [1.68, 5.74], respectively. Notably, TCEs lasting < 12 weeks yielded the most substantial improvement in UPDRS-III scores, whereas TCEs lasting ≥12 weeks produced the most significant enhancement in TUGT and BBS scores (Table 2).

**Table 2:** Subgroup analysis of UPDRS-III, TUGT and BBS outcome indicators.

**Variable**		**Motor symptoms**					**Mobility**					**Mobility**				
		**UPDRS-III**					**TUGT**					**BBS**				
		***S***	***N***	***I*** ^2^	**MD (95% CI)**	***P***	***S***	***N***	***I*** ^2^	**MD (95% CI)**	**P**	**S**	***N***	***I*** ^2^	**MD (95% CI)**	***P***
Exercise duration	≥12 week	7	203/203	59%	−4.28 [−5.99, −2.58]	< 0.00001	5	128/124	72%	−3.81 [−5.19, −2.43]	< 0.00001	2	72/74	0%	3.75 [2.05, 5.45]	< 0.0001
	< 12 week	4	99/94	89%	−4.79 [−7.66, −1.93]	0.001	3	118/120	21%	−1.32 [−2.12, −0.52]	0.001	5	166/166	88%	3.71 [1.68, 5.74]	0.0003
Exercise frequency	≥3 times/week	6	151/149	82%	−4.24 [−6.29, −2.19]	< 0.0001	5	173/176	89%	−2.68 [−4.63, −0.72]	=007	6	203/205	85%	3.71 [1.85, 5.56]	< 0.0001
	< 3 times/week	5	151/148	59%	−4.67 [−6.72, −2.62]	< 0.00001	3	73/68	39%	−3.04 [−4.11, −1.96]	< 0.00001	1	35/35		3.73 [1.84, 5.62]	= 0.0001
Duration session	≥60 min	9	256/251	76%	−4.86 [−6.57, −3.14]	< 0.00001	7	181/179	81%	−3.05 [−4.38, −1.73]	< 0.00001	5	188/190	88%	4.16 [1.37, 6.94]	0.003
	< 60 min	2	46/46	0%	−2.10 [−2.97, −1.23]	< 0.00001	1	65/65	85%	−1.12 [−2.18, −0.06]	0.04	2	50/50	0%	3.00 [2.19, 3.81]	< 0.00001
Type of exercise	Taichi	6	188/187	83%	−3.79 [−5.89, −1.69]	=0.0004	4	131/128	84%	−3.41 [−5.00, −1.83]	< 0.0001	4	131/133	82%	5.03 [2.08, 7.97]	=0.0008
	Qigong	5	114/110	11%	−5.54 [−8.81, −2.27]	0.0009	4	115/116	58%	−1.82 [−3.21, −0.43]	=0.01	3	107/107	68%	2.29 [0.93, 3.65]	=0.001
Control group type	Active-control	6	181/178	81%	−4.46 [−6.55, −2.36]	< 0.0001	3	115/115	82%	−2.62 [−4.52, −0.72]	=0.007	4	131/131	63%	2.61 [1.47, 3.75]	< 0.00001
	Non-active control	5	121/119	84%	−4.27 [−7.48, −1.06]	=0.009	5	131/129	86%	−2.90 [−4.75, −1.06]	=0.002	3	107/109	85%	5.52 [0.98, 10.07]	=0.02
Duration of disease	≥3 years	8	237/232	66%	−5.58 [−7.44, −3.73]	< 0.00001	7	223/226	85%	−2.89 [−4.33, −1.44]	< 0.00001	4	143/145	86%	4.94 [2.25, 7.62]	0.0003
	< 3 years	3	65/65	73%	−2.32 [−4.08, −0.56]	0.01	1	23/18		−2.17 [−3.46, −0.88]	0.0010	3	95/95	33%	2.01 [0.73, 3.29]	0.002

#### 3.5.2 Exercise frequency

The exercise frequency involved in the experiments included in this study was mainly 1 time/day, 2 times/week, 3 times/week, 5 times/week and 10 times/week, and WHO recommends 3 or more times/week of exercise for older adult people aged 65 years or older (Bull et al., 2020),and since the mean age of the experimental group of the subjects included in the present study was 65.15 years old, and the mean age of the control group was 65.00 years old, the threshold for the subgroups of the frequency of intervention was 3 times/week. The results of subgroup analysis showed that both < 3 times/week and ≥ 3 times/week TCE improved UPDRS-III scores in PD patients, with the greatest effect brought by TCE less than 3 times/week, with an MD=-4.67,95% CI [−6.72, −2.62], followed by ≥ 3 times/week, with an MD = −4.24,95% CI [−6.29, −2.19]; TCE with an exercise frequency of < 3 times/week produced the greatest effect in improving TUGT in patients with PD, with an MD = −3.04, 95% CI [−4.11, −1.96], followed by ≥3 times/week, with an MD=-2.68, 95% CI [−4.63, −0.72]; and TCE performed less than 3 times/week produced the greatest effect size in improving BBS scores in PD patients, with an MD = 3.73,95% CI [1.84, 5.62], followed by ≥3 times/week, with an MD = 3.71,95% CI [1.85, 5.56].

#### 3.5.3 Duration session

The most prevalent duration for exercise workouts was 60 min, with the 14 studies encompassing various single exercise durations of 60 min, 30–45 min, 30 min, 40 min, and 90 min, subsequently categorized into two subgroups: ≥60 min and < 60 min. Subgroup analyses revealed that exercise durations of ≥60 min and < 60 min significantly influenced improvements in UPDRS-III scores in Parkinson's disease patients, with MD = −4.86 (95% CI [−6.57, −3.14]) and −2.10 (95% CI [−2.97, −1.23]), respectively; enhancements in TUGT scores, with respective MD = −3.05 (95% CI [−4.38, −1.73]) and −1.12 (95% CI [−2.18, −0.06]); and improvements in BBS scores, with MD = 4.16 (95% CI [1.37, 6.94]) and 3.00 (95% CI [2.19, 3.81]), respectively. The conventional gong practice, with a single exercise duration of 60 min or more, yielded the most significant impact on UPDRS-III, TUGT, and BBS scores.

### 3.6 Subgroup analysis of variables influencing intervention effectiveness

#### 3.6.1 Type of exercise

This study primarily focused on Tai Chi and Qigong as the principal forms of exercise therapy. The findings indicated that Qigong exercise yielded the most significant improvement in UPDRS-III scores among PD patients (MD = −5.54, 95% CI [−8.81, −2.27]), followed by Tai Chi with an MD = −3.79 (95% CI [−5.89, −1.69]). Additionally, Tai Chi demonstrated the most substantial effect on enhancing TUGT scores in PD patients (MD = −3.41, 95% CI [−5.00, −1.83]), the next is the Qigong exercise MD = −1.82, 95%CI [−3.21, −0.43]. The Tai Chi exercise demonstrated the most significant improvement in BBS scores for patients with Parkinson's Disease, with a mean difference MD = 5.03,95% CI [2.08, 7.97]. This was followed by Qigong exercise, which yielded an MD = 2.29,95% CI [0.93, 3.65] (Table 2).

#### 3.6.2 Control group type

Research indicates that Shen et al. (2016), the type of control group influences the magnitude of intervention effects. In this study, control groups were classified into active and passive interventions. Subgroup analyses revealed that the effect size of TCE on UPDRS-III scores in Parkinson's disease patients was superior in the active intervention group (MD = −4.46, 95% CI [−6.55, −2.36]) compared to the passive intervention group. Conversely, the passive intervention group demonstrated better TUGT scores (MD = −2.90, 95% CI [−4.75, −1.06]) than the active intervention group (MD of −2.62, 95% CI [−4.52, −0.72]). Additionally, the passive intervention group outperformed the active intervention group on BBS scores (MD = 5.52, 95% CI [0.98, 10.07] vs. MD = 2.61, 95% CI [1.47, 3.75]).

#### 3.6.3 Duration of disease

Considering the trajectory of illness advancement in Parkinson's disease and findings from clinical research, a duration criterion of 3 years was established for grouping purposes. The findings of the subgroup analysis indicated that disease duration of ≥3 years and < 3 years significantly influenced UPDRS-III scores in Parkinson's disease patients (MD = −5.58, 95% CI [−7.44, −3.73] and −2.32, 95% CI [−4.08, −0.56], respectively); TUGT scores, MD = −2.89, 95% CI [−4.33, −1.44] and −2.17, 95% CI [−3.46, −0.88], respectively; and BBS scores with MD = 4.94, 95% CI [2.25, 7.62] and 2.01, 95% CI [0.73, 3.29], respectively. The TCE intervention was the most efficacious for PD patients with a disease duration of 3 years or more.

### 3.7 Meta-regression

In the meta-regression analysis, the encoding processing was as follows: exercise duration: < 12 weeks is encoded as 1, ≥12 weeks is encoded as 2; exercise frequency: < 3 times/week is coded as 1, and ≥ 3 times/week is coded as 2; duration session: < 60 min is encoded as 1, and ≥ 60 min is encoded as 2 (Table 3).

**Table 3:** Meta-regression.

**Variable**	**Motor symptoms**			**Mobility**					
	**UPDRS-III**			**TUGT**			**BBS**		
	**Coefficient**	***P***	**95%CI**	**Coefficient**	***P***	**95%CI**	**Coefficient**	***P***	**95%CI**
Duration of intervention (weeks)	1.349427	0.182	[−0.8379019, 3.536756]	0.7100188	0.165	[−0.5266564, 1.946694]	−1.052887	0.083	[−2.446714, 0.3409399]
Exercise frequency (session per week)	0.3366438	0.649	[−1.381405, 2.054692]	−0.2752495	0.510	[−1.44899, 0.8984914]	−0.4887727	0.290	−1.963001, 0.9854553
Session duration (min)	−0.9963718	0.278	[−3.040279, 1.047535]	0.0780238	0.879	[−1.427056, 1.583103]	−0.2826235	0.409	[−1.45529, 0.8900427]

The meta-regression analysis of TCE dose intervention for motor symptoms and mobility in PD patients revealed that exercise duration, exercise frequency, and session duration did not significantly impact the UPDRS-III, TUGT, and BBS indices. *P* > 0.05, 95% confidence interval includes 0.

### 3.8 Publication bias

Upon examining the funnel plot and identifying a symmetrical distribution of the study sites, it was first determined that the likelihood of publication bias was absent. A supplementary Egger test was conducted to validate this assessment. The analysis indicated an absence of publication bias in the studies concerning UPDRS-III (regression intercept = −2.540148, *P* = 0.401 > 0.05), TUGT (regression intercept = −3.010592, *P* = 0.341 > 0.05), and BBS (regression intercept = 7.157901, *P* = 0.203 > 0.05), the three outcome measures.

Sensitivity analyses were conducted utilizing the merged model alteration method, and the item-by-item exclusion technique demonstrated that all study effect sizes exhibited stable distribution. This outcome demonstrated that the removal of any single study or many studies did not substantially influence the overall combined effect size, hence confirming the stability and credibility of the meta-analysis conclusions in this study.

## 4 Discussion

### 4.1 TCE for PD patients

The meta-analysis revealed that the effect sizes (MD) of the three indicators of motor symptoms and mobility for TCE intervention in PD patients were statistically significant. To enhance its application in clinical practice, the effect sizes were juxtaposed with the Minimum Clinically Important Difference (MCID). The MCID value for PD patients was established at −3.25 points (UPDRS-III) (Horváth et al., 2015) using the anchoring approach (Bloom et al., 2023) although definitive values for the MCID of TUGT and BBS in PD patients remain unavailable. In clinical practice, patients can observe a decrease in motor symptoms (MD = −4.38 for UPDRS-III), while the status of functional walking and balance, as perceived by patients, remains uncertain.

Meta-analysis shows that optimal outcomes for motor symptoms and mobility were attained with TCE sessions duration ≥60 min, exercise frequency < 3times/week particularly in patients with a disease duration of more than 3 years. Subgroup analyses indicated that Qigong exercise, exercise duration < 12 weeks, exercise frequency < 3 times/week, with each session duration ≥ 60 min, yielded optimal results in alleviating motor symptoms in Parkinson's disease patients. Conversely, for enhancing mobility, Tai Chi exercise, exercise duration ≥ 12 weeks, exercise frequency < 3 times/week, session duration also ≥ 60 min, demonstrated the most significant efficacy. Our research indicated that TCE intervention yielded the most significant results in PD patients with an illness duration of three years or longer, and that the intervention modality in the control group also influenced the intervention's efficacy. Consequently, our review suggests that it is advisable for future research to enhance the emphasis on the study population and the intervention method utilized in the control group.

### 4.2 TCE for motor symptoms

The third section of the Unified Parkinson's Disease Rating Scale (UPDRS) serves as the primary criterion for physicians to assess patients‘ motor symptoms, comprising 18 items including gait, posture, resting tremor, and myotonia, among others, with elevated scores indicating more severe motor function problems. A higher score indicates more severe motor function issues. The slow pace of Qigong, a vital aspect of aerobic exercise, can improve cerebellar regulation of movement timing and suppress excessive inhibitory output from the basal ganglia (Xiao et al., 2016). Activation of the nigrostriatal pathway via rhythmic exercise can augment dopamine release from the striatum, and this sensory-motor integration improves the connectivity between the cerebellum and the motor cortex, thereby optimizing motor programming (Hirsch et al., 2016) and mitigating motor symptoms. Research indicates that Petzinger et al. (2013) aerobic exercise lasting 12 weeks or less can enhance the density of striatal D2 receptors; however, extending the duration to 24 weeks may lead to receptor desensitization. Clinical trials have demonstrated (Li et al., 2012) a decline in participants' adherence to TCE interventions beyond 12 weeks, highlighting that the efficacy of TCE interventions on the motor symptoms of patients with PD is influenced by multiple factors, and that prolonged intervention does not necessarily yield superior outcomes.

### 4.3 TCE for mobility

The Timed Up and Go Test (TUGT) is an instrument principally utilized to assess the functional ambulation capacity and fall risk in people with Parkinson's disease. The assessment evaluates walking proficiency, wherein the participant begins seated, thereafter rises, ambulates, pivots, and returns to a seated position. Three measurements are recorded and averaged; a shorter duration signifies superior walking abilities, whereas a length of ≥5 seconds indicates a slower stride and diminished balance, and a duration of ≥12 seconds denotes a heightened danger of falling. The Berg Balance Scale (BBS) assesses the balance capabilities of patients with Parkinson's disease (PD). The scale comprises 14 items, such as standing up, sitting down, and independently standing and sitting. Elevated scores signify superior balance. Prolonged Tai Chi training over 12 weeks can activate the cerebellar-red nucleus pathway, enhance the centrifugal contraction of the quadriceps muscle, and improve core muscle coordination, thereby increasing the speed of vestibulospinal reflexes, reducing turnaround time, and minimizing the number of turning steps through continuous weight shifting and multiaxial rotation (Li et al., 2012; Amano et al., 2013). Extended physical activity increases connections between the cerebellum and the prefrontal brain, hence strengthening postural prediction and improving walking functionality; dynamic postural balance can be augmented by stimulating pathways between the cerebellar hemispheres and the motor cortex (Fan et al., 2020; Herman et al., 2014).

This meta-analysis demonstrates that the ideal frequency for Qigong or Tai Chi is < 3times/week, with each session duration ≥ 60 min; this regimen is more successful in addressing motor symptoms and mobility in patients with a disease duration of more than 3 years. The rationale is because in the initial stages of the disease, patients exhibit symptoms like tremor and moderate ankylosis, which minimally impact their daily activities, resulting in a limited efficacy of TCE intervention. Low-frequency training prevents dopamine receptor desensitization, preserves D2 receptor sensitivity (Petzinger et al., 2013). Conducting sessions spaced approximately 48 hours apart (as in low frequency regimens) may further enhance BDNF-mediated synaptic remodeling. In the practical application of TCE, its motor structure is not isolated but comprises a “chain of actions.” A complete stimulus effect necessitates sufficient time, exceeding 60 min, to accomplish the entire chain, ensuring a full sequence of 2–3 rounds. Insufficient duration may hinder the formation of stable neuromuscular memory.

The heterogeneity of this meta-analysis may arise from the absence of clear diagnostic criteria for Parkinson's disease (PD) in some included studies, as well as the lack of differentiation between PD subtypes. This may exacerbate the variability in responses to exercise due to the differing severity of motor symptoms among participants. In conclusion, Parkinson's disease is a progressive neurological disorder that can be affected by numerous circumstances.

There are some advantages of this meta-analysis. The PRISMA guidelines and the Cochrane Handbook were strictly followed, and the review methodology was registered. Eight Chinese and English databases were searched, 14 RCTs were included, five studies published in 2022–2024 were included compared to the study by Wu et al. (2022) and Xiao et al. (2016), the data were more current, and our review included different types of TCEs (e.g., Wuqinxi). Our review focuses on clinical utility, provides the best exercise choices for PD patients with different rehabilitation goals, and systematically analyzes the impact of disease duration on the effectiveness of TCE interventions, optimizes the selection of intervention populations, and provides a direct basis for the clinical development of a “personalized exercise prescription”; in this review, we also found that In this review, we also found that there is a difference in the effect of the type of control group on the efficacy of TCE. This is the first time that the MCID is used to assess the intervention effect, which is directly related to the actual benefit perceived by patients in clinical practice, all of which improve the comprehensiveness and scientificity of our review.

This meta-analysis presents several advantages. The PRISMA standards and the Cochrane Handbook were meticulously adhered to, and the review technique was duly registered. Eight Chinese and English databases were examined, resulting in the inclusion of 14 randomized controlled trials (RCTs), with five studies published between 2022 and 2024 compared to the research conducted by Wu et al. (2022), The data were more recent, and several forms of TCEs (e.g., Wuqinxi) were incorporated in the research included. Our evaluation emphasizes clinical utility by quantifying the appropriate program to deliver the most effective exercise options for PD patients with varying rehabilitation objectives (for example, the optimal exercise dose for improving patients' motor symptoms is: Qigong exercise, < 12 weeks, < 3 times/week, and ≥60 min), whereas the study by Wu et al. validated the overall efficacy of TCE, detailing the relationship between dosage and effect. This review systematically evaluated the impact of disease duration on the efficacy of TCE intervention, refined the selection criteria for the intervention population, and established a direct foundation for the clinical advancement of “personalized exercise prescription”; additionally, we identified that the type of control group influenced the effectiveness of TCE differently. For the first time, the MCID was employed to evaluate the intervention impact, directly correlating with the tangible benefits experienced by patients in clinical practice, these advantages facilitate the enhancement of the comprehensiveness and scientific rigor of our review.

This meta-analysis mostly exhibits the following limitations: (1) The quantity of included studies is inadequate, and the danger of bias is comparatively elevated. For example,the inability of any included research to implement double-blind procedures, coupled with only two studies reporting allocation concealment, will undermine the stability and trustworthiness of the results. The conclusion requires validation by additional rigorous studies;(2) when patients with Parkinson's Disease engage in exercises with TCE, variations in the type of exercise, exercise duration, exercise frequency, and specific intervention protocols may influence the study outcomes; (3) We cannot compare the effect size with the MCID in the TUG and BBS domains due to ongoing studies examining the MCID in PD patients. Additional study is required to evaluate the Minimal Clinically Important Difference (MCID) of functional walking ability and balance function to enhance comprehension of the true efficacy of therapeutic strategies for Parkinson's disease patients;(4) the limited number of outcome indicators in this study presents constraints in comprehensively assessing the motor symptoms and mobility of Parkinson's Disease patients; (5) the follow-up duration was relatively brief, rendering it impossible to validate the long-term effects of TCE on the amelioration of motor symptoms and mobility in PD patients. Future research are advised to implement a uniform TCE intervention strategy and prolong the follow-up duration to investigate its long-term effects on motor symptoms in PD patients.

## 5 Conclusion

This meta-analysis demonstrated that TCE enhances motor symptoms, functional walking ability, and balance in Parkinson's disease patients, as indicated by improvements in UPDRS-III, TUGT, and BBS scores. In comparison to the MCID, patients with Parkinson's disease can recognize the alleviation of motor symptoms. Further research on the Minimal Clinically Important Difference(MCID)regarding the activity capabilities of Parkinson's Disease (PD) patients is essential for a more accurate assessment of intervention efficacy in this population. The ideal exercise regimen for TCE to address motor symptoms and mobility in individuals with Parkinson's disease < 3times/week, with each session duration ≥60 min. The results require additional verification due to the potential for bias.

## Data Availability

The original contributions presented in the study are included in the article/Supplementary material, further inquiries can be directed to the corresponding author.
