# Supplementary material for: Traditional Chinese exercises for motor symptoms and mobility in patients with Parkinson's disease: a systematic review and meta-analysis
Source: Front Aging Neurosci. 2025 Aug 19;17:1612913. doi: 10.3389/fnagi.2025.1612913 (PMC12401968; doi:10.3389/fnagi.2025.1612913)
Supplement: Supplementary file 1 [file Supplementary_file_1.docx]

***Supplementary Material***

**Supplementary Figures**

**Table 1**

| Exercise | Definition | Philosophical background | Movement characteristics | Correlation with PD treatment | References |
| --- | --- | --- | --- | --- | --- |
| Tai chi | The ancient Chinese internal martial arts, which combine slow movements, breath control and meditation | The ancient Chinese internal martial arts, which combine slow movements, breath control and meditation | Slow and continuous arc-shaped movements, center of gravity shift, mainly standing practice, emphasizing the coordination of body and mind | Improve balance function (reduce the risk of falls), gait stability, lower limb muscle strength and quality of life, and relieve movement symptoms | [1; 2] |
| Yijinjing | Traditional guiding exercises aim to "transform the tendons and bones", strengthening the tendons, bones and internal organs | Originating from the Chan School of Buddhism and the meridian theory of traditional Chinese medicine; Emphasize the unity of "form, qi and spirit" | The Twelve postures combine dynamic and static movements, with stretching and twisting as the main approaches, complemented by specific breathing techniques, and focus on tendon stretching | Enhance muscle strength and joint flexibility, improve posture control, and have potential neuroprotective effects | [3; 4; 5] |
| Wuqinxi | The guiding techniques imitating five animals: the tiger, the deer, the bear, the ape and the bird | It was created by Hua Tuo, a renowned physician of the Han Dynasty, based on the theory of "bionics" in traditional Chinese medicine and the correspondence between the five Elements and the internal organs | Bionic dynamic practice, with movements stretching and imitating shapes, emphasizing the twisting of the waist and spine, and having a light and natural rhythm | Enhance coordination and joint range of motion, improve balance, and regulate emotions | [6; 7; 8; 9] |
| Liuzijue | A breathing exercise that regulates the internal organs through six types of vocalization (xu, he, hu, si, chui, and xi4) | According to the theory of "five tones matching five internal organs" in traditional Chinese medicine, breathing regulates the ascending and descending of qi and muscles | The main practice is standing or sitting meditation, combined with breathing exercises, pronunciation and simple gesture guidance, emphasizing the depth of breathing | Improve respiratory function, clarity of speech and autonomic nerve regulation, and relieve constipation | [10; 11] |
| Baduanjin | The classic guiding exercises composed of eight movements emphasize "guiding qi to achieve harmony and pull-ups to make the body gentle". | Integrating the thoughts of Confucianism, Taoism and medicine, it emphasizes the combination of movement and stillness, and the co-cultivation of form and spirit | Symmetrical standing exercises, with gentle and smooth movements, combined with breathing and concentration, structured and standard | Significantly improve balance ability, walking ability and quality of life, and alleviate bradykinesia and stiffness | [12; 13] |

**Supplementary Appendix – Electronic searches**

**Pubmed**

((((((((((((((Parkinson Disease) OR (parkinsonism)) OR (Parkinson)) OR (Idiopathic Parkinson's Disease)) OR (Lewy Body Parkinson's Disease)) OR (Parkinson's Disease, Idiopathic)) OR (Parkinson's Disease, Lewy Body)) OR (Paralysis Agitans)) OR (Parkinson's Disease)) OR (Idiopathic Parkinson Disease)) OR (Lewy Body Parkinson Disease)) OR (Primary Parkinsonism)) OR (Parkinsonism, Primary)) OR (Parkinson Disease, Idiopathic)) AND ((((((((((((((((Tai Ji) OR (Tai-ji)) OR (Tai Chi)) OR (Chi, Tai)) OR (Tai Chi Chuan)) OR (Taiji)) OR (Taijiquan)) OR (T'ai Chi)) OR (Tai Ji Quan)) OR (Ji Quan, Tai)) OR (Quan, Tai Ji)) OR (((Qigong) OR (Ch'i Kung)) OR (Qi Gong))) OR (Yijinjing)) OR (Wuqinxi)) OR (Liuzijue)) OR ((((((((Baduanjin) OR (baduan jin)) OR (ba duan jin)) OR (eight section brocades)) OR (brocades)) OR (eight trigrams boxing)) OR (eight-treasured exercises)) OR (eight pieces road)))

**Web of Science**

((((((((((TS=(Tai Ji)) OR TS=(Tai-ji)) OR TS=(Tai Chi)) OR TS=(Chi, Tai)) OR TS=(Tai Chi Chuan)) OR TS=(Taiji)) OR TS=(Taijiquan)) OR TS=(T'ai Chi)) OR TS=(Tai Ji Quan)) OR TS=(Ji Quan, Tai)) OR TS=(Quan, Tai Ji) OR (((TS=(Qigong)) OR TS=(Ch'i Kung)) OR TS=(Qi Gong)) OR TS=(Yijinjing) OR TS=(wuqinxi) OR TS=(Liuzijue) OR (((((((TS=(Baduanjin )) OR TS=(Baduan jin)) OR TS=(Ba duan jin)) OR TS=(eight section brocades)) OR TS=(brocades)) OR TS=(eight trigrams boxing )) OR TS=(eight-treasured exercises)) OR TS=(eight pieces road) AND (((((((((((((TS=(Parkinson Disease)) OR TS=(Parkinson)) OR TS=(parkinsonism)) OR TS=(Idiopathic Parkinson's Disease)) OR TS=(Lewy Body Parkinson's Disease)) OR TS=(Parkinson's Disease, Idiopathic)) OR TS=(Parkinson's Disease, Lewy Body)) OR TS=(Paralysis Agitans)) OR TS=(Parkinson's Disease)) OR TS=(Idiopathic Parkinson Disease)) OR TS=(Lewy Body Parkinson Disease)) OR TS=(Primary Parkinsonism)) OR TS=(Parkinsonism, Primary)) OR TS=(Parkinson Disease, Idiopathic)

**Cochrane library**

(((Tai Ji)MeSH OR (Tai-Ji):ti,ab OR (Tai Chi):ti,ab OR (Chi Tai):ti,ab OR (Tai Ji Quan):ti,ab OR (Quan Tai Ji):ti,ab OR (Ji Quan Tai):ti,ab OR (Taiji):ti,ab OR (Taijiquan):ti,ab OR (T'ai Chi):ti,ab OR (Tai Chi Chuan):ti,ab OR (Qigong)MeSH OR (Qi Gong):ti,ab OR (Chi Kung):ti,ab OR (yijinjing):ti,ab OR (wuqinxi):ti,ab OR ((Baduanjin):ti,ab OR (baduan jin):ti,ab OR (ba duan jin):ti,ab OR (eight section brocades):ti,ab OR (brocades):ti,ab OR (eight trigrams boxing):ti,ab OR (eight-treasured exercises):ti,ab OR (eight pieces road):ti,ab AND ((Parkinson's Disease):ab,ti OR (Idiopathic Parkinson's Disease):ab,ti OR (Lewy Body Parkinson's Disease):ab,ti OR (Parkinson's Disease, Idiopathic):ab,ti OR (Parkinson's Disease, Lewy Body):ab,ti OR (Parkinson Disease, Idiopathic):ab,ti OR (Idiopathic Parkinson Disease):ab,ti OR (Lewy Body Parkinson Disease):ab,ti OR (Primary Parkinsonism):ab,ti OR (Parkinsonism, Primary):ab,ti OR (Paralysis Agitans):ab,ti)

**EBSCO**

TX Tai ji OR TX Tai Chi OR TX Chi, Tai OR TX Tai Chi Chuan OR TX Taiji OR TX Taijiquan OR TX T'ai Chi OR TX Tai-Ji Quan OR TX Ji Quan, Tai OR TX Quan, Tai Ji OR TX Qigong OR TX Qigong OR TX Health Qigong OR TX Ch'i Kung OR TX Qi Gong OR TX Yijinjing OR TX Wuqinxi OR TX Liuzijue OR TX Baduanjin OR TX baduan jin OR TX ba duan jin OR TX eight section brocades OR TX brocades OR TX eight trigrams boxing OR TX eight-treasured exercises OR TX eight pieces road AND TX Parkinson Disease OR TX Parkinson OR TX parkinsonism OR TX Paralysis Agitans OR TX Parkinson's Disease OR TX Primary Parkinsonism OR TX Parkinsonism, Primary OR TX Idiopathic Parkinson's Disease OR TX Lewy Body Parkinson's Disease OR TX Parkinson's Disease, Idiopathic OR TX Parkinson's Disease, Lewy Body OR TX Idiopathic Parkinson Disease

**Table 2**

| study/evaluation item | Random sequence generation (selection bias) | Allocation concealment (selection bias) | Blinding of participants and personnel (performance bias) | Blinding of outcome assessment (detection bias) | Incomplete outcome data (attrition bias) | Selective reporting (reporting bias) | Other bias |
| --- | --- | --- | --- | --- | --- | --- | --- |
| X-L. Liu et al 2017 | Unclear risk | Unclear risk | Unclear risk | Unclear risk | Low risk | Low risk | High risk |
| H. Chen et al 2024 | Low risk | Unclear risk | Unclear risk | Unclear risk | Low risk | Low risk | Low risk |
| Y. You et al 2020 | Low risk | Unclear risk | Unclear risk | Unclear risk | Low risk | Low risk | Low risk |
| Y. Zhu et al 2011 | Low risk | Unclear risk | High risk | Unclear risk | Low risk | Low risk | Unclear risk |
| L. Ding et al 2021 | Unclear risk | Unclear risk | Unclear risk | Low risk | Unclear risk | Low risk | Low risk |
| H-H. Cao et al 2021 | Low risk | Unclear risk | Unclear risk | Unclear risk | Low risk | Low risk | Unclear risk |
| X-Z. Kong et al 2022 | Low risk | Unclear risk | Unclear risk | Unclear risk | Low risk | Low risk | Low risk |
| Z. Li et al 2022 | Low risk | Low risk | High risk | Low risk | Low risk | Low risk | Unclear risk |
| C-L Chang et al 2024 | Unclear risk | Unclear risk | Unclear risk | Unclear risk | Low risk | Low risk | Low risk |
| K-F Li et al 2024 | Low risk | Unclear risk | High risk | Low risk | Low risk | Low risk | Unclear risk |
| X. Li et al 2021 | Unclear risk | Unclear risk | Unclear risk | Unclear risk | Unclear risk | Low risk | High risk |
| Q. Gao et al 2014 | Unclear risk | Low risk | High risk | Low risk | Low risk | Low risk | Unclear risk |
| F. Li et al 2012 | Low risk | Unclear risk | Unclear risk | Low risk | Low risk | Low risk | High risk |
| G. Vergara Diaz et al 2017 | Low risk | Unclear risk | Unclear risk | Low risk | Low risk | Low risk | High risk |

**Subgroup analysis of exercise dosage**

Exercise duration(order is: UPDRS-Ⅲ、TUGT、BBS)


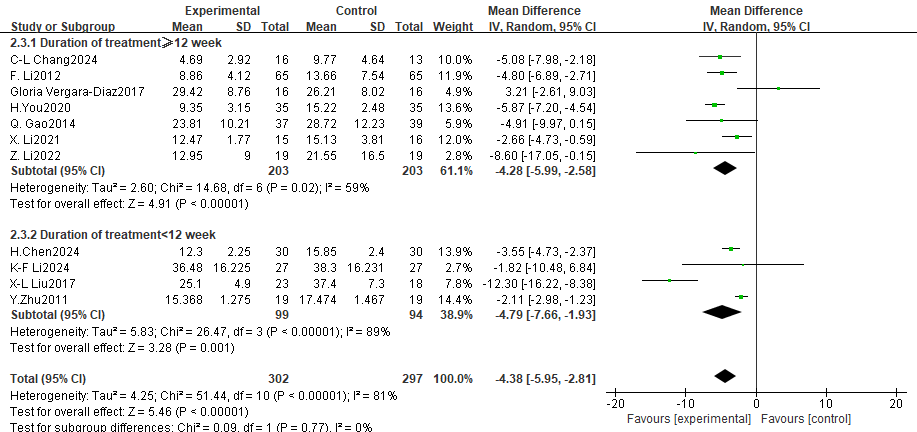


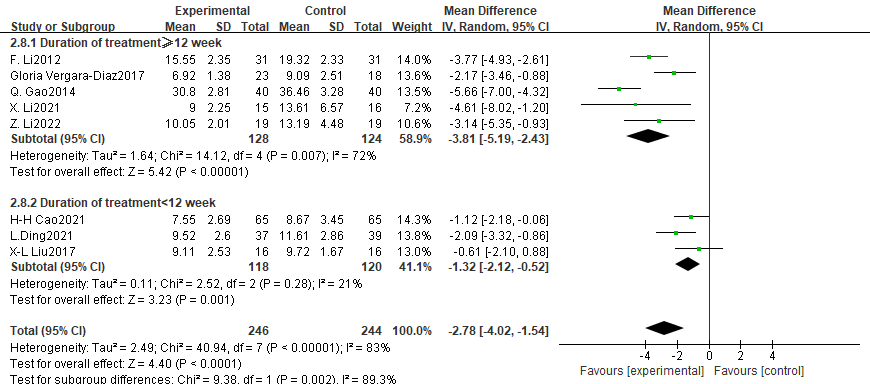


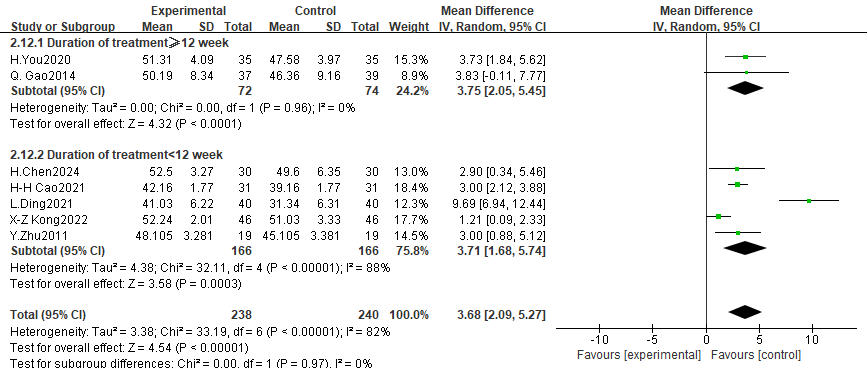


Exercise frequency：


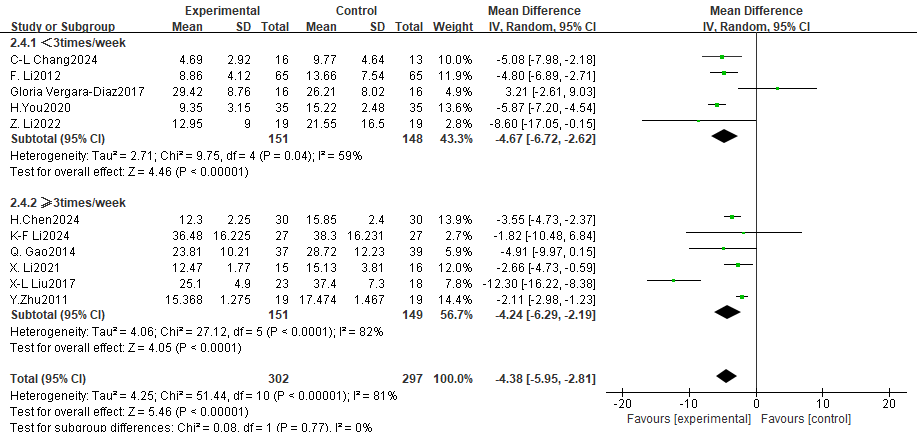


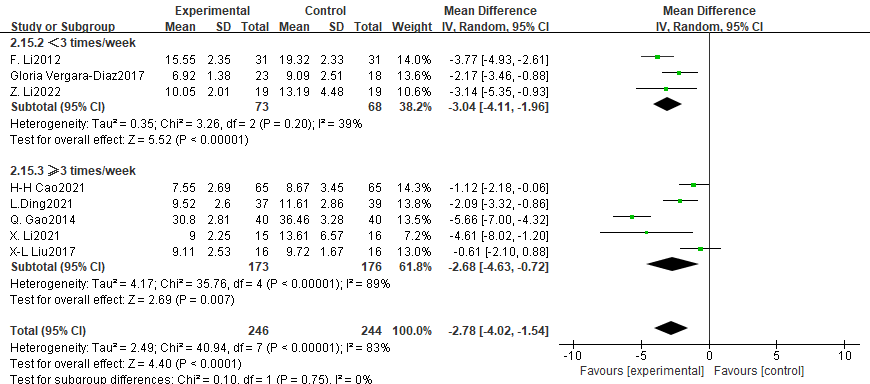


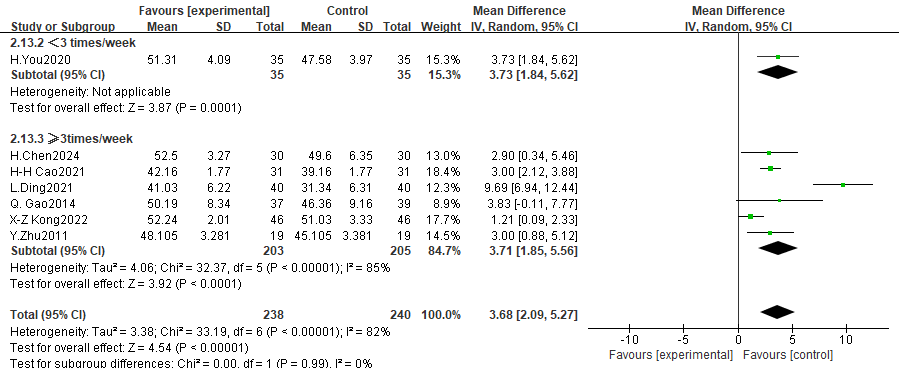


Duration session：


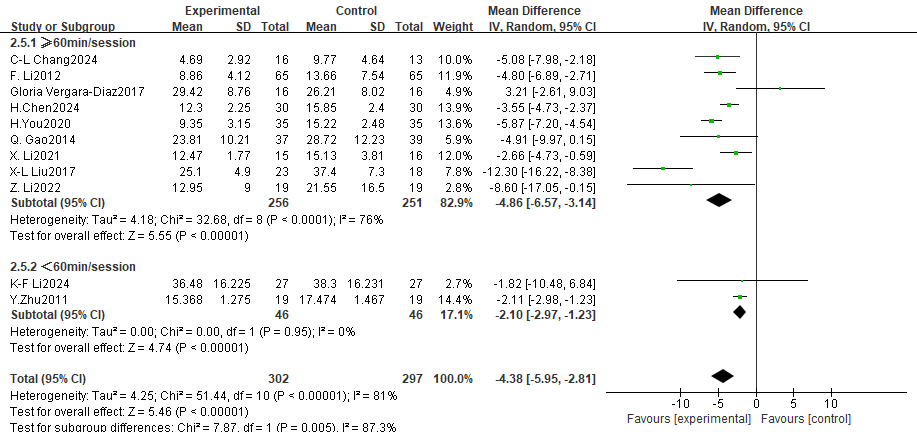


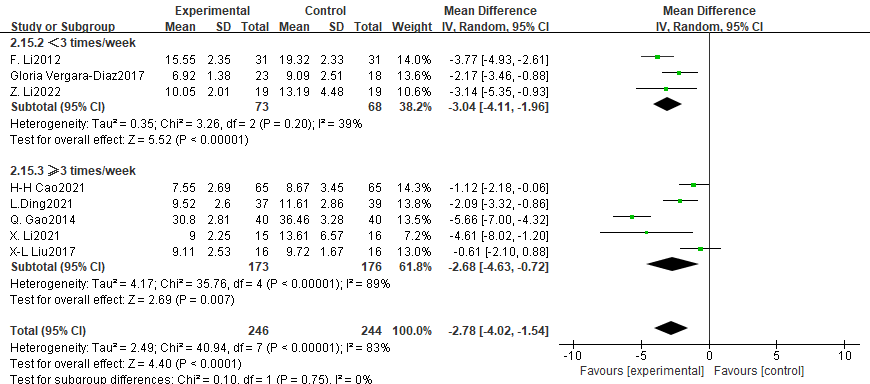


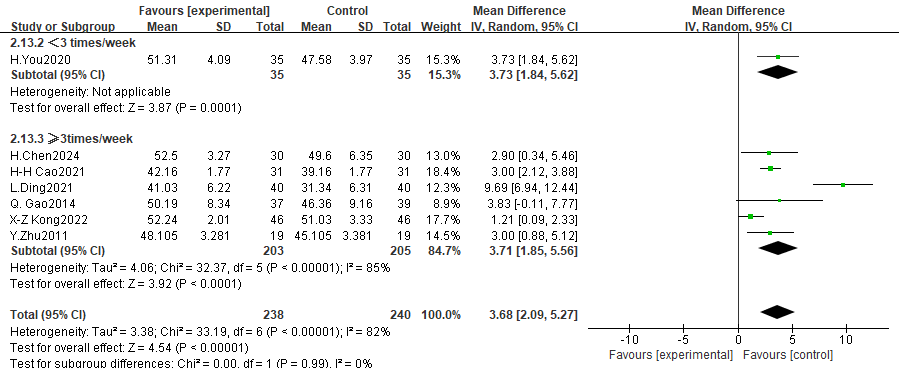


Type of exercise：


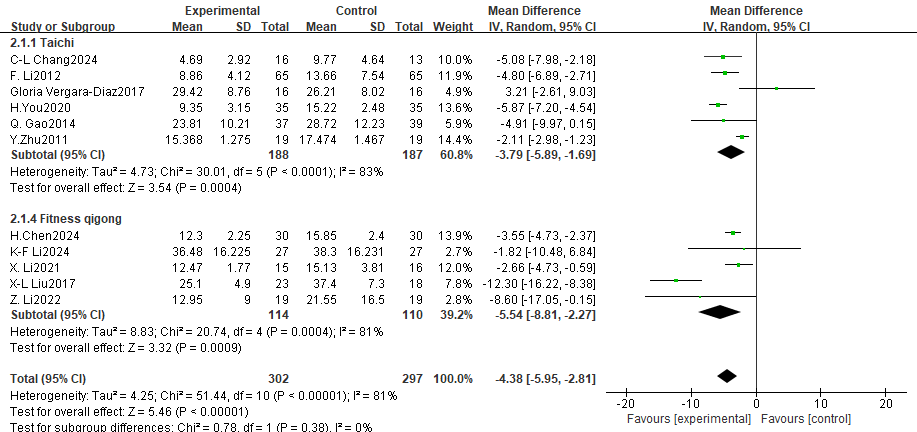


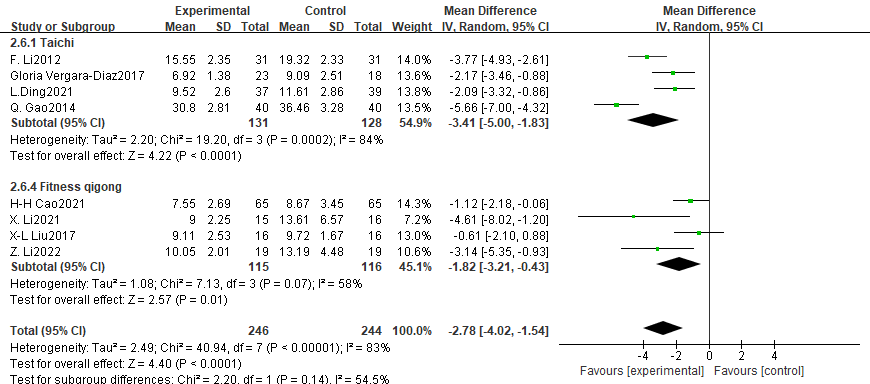


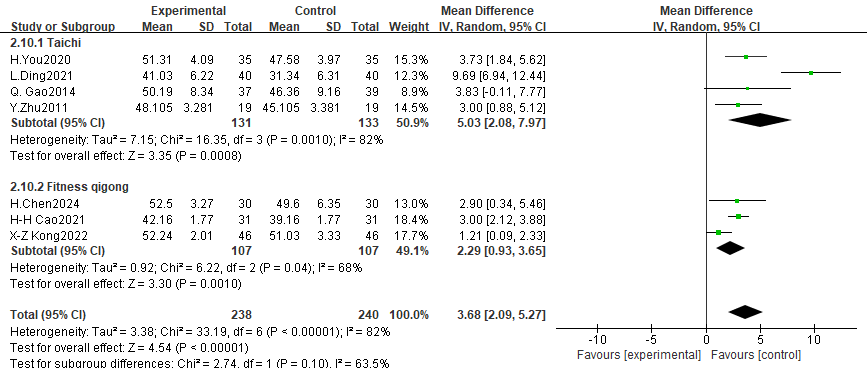


Control group type：


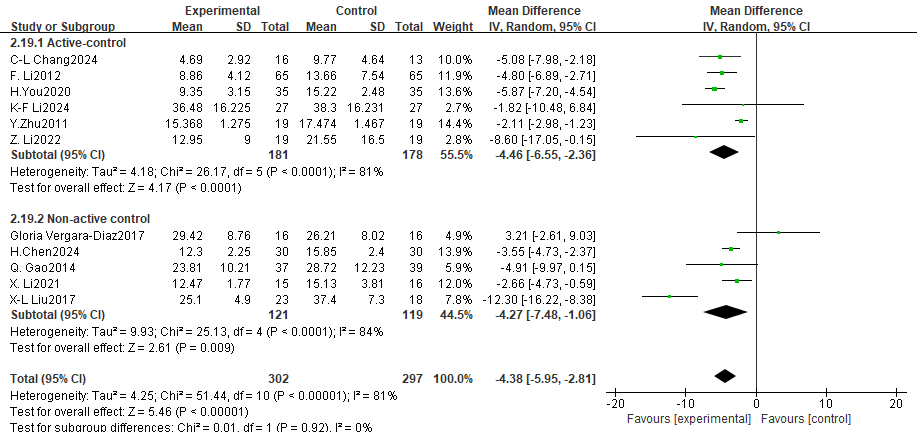


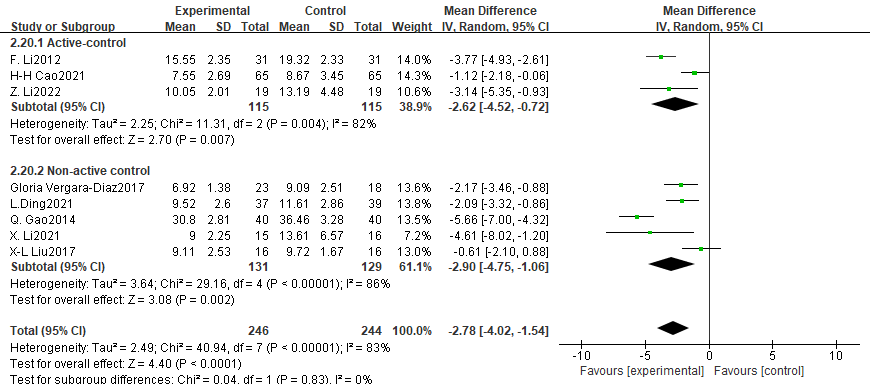


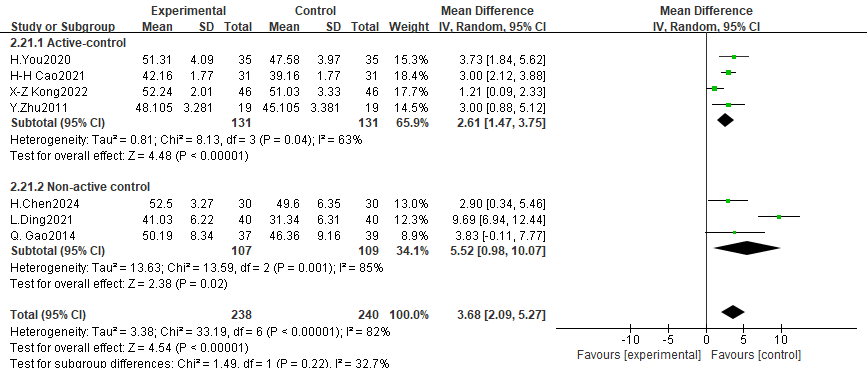


Duration of disease：


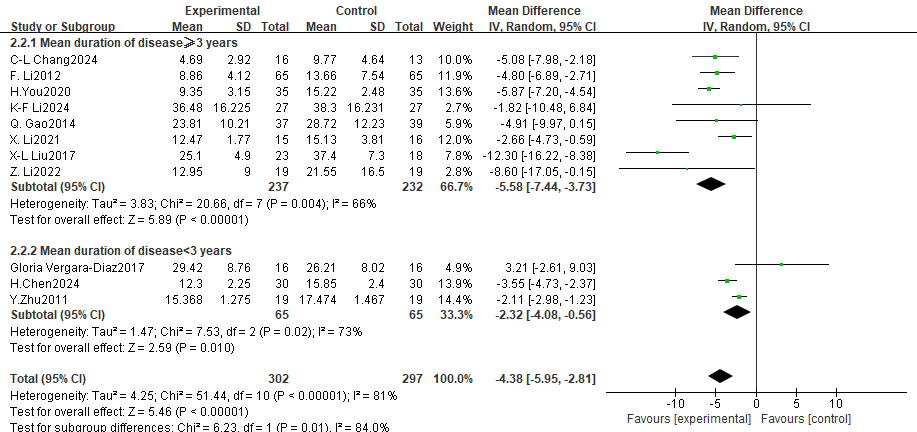


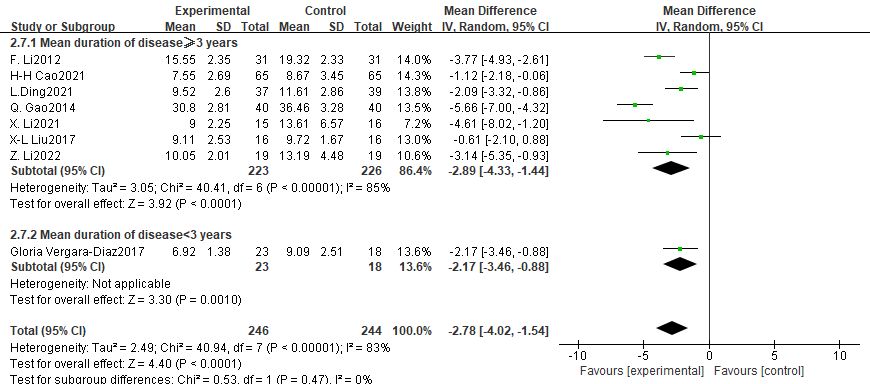


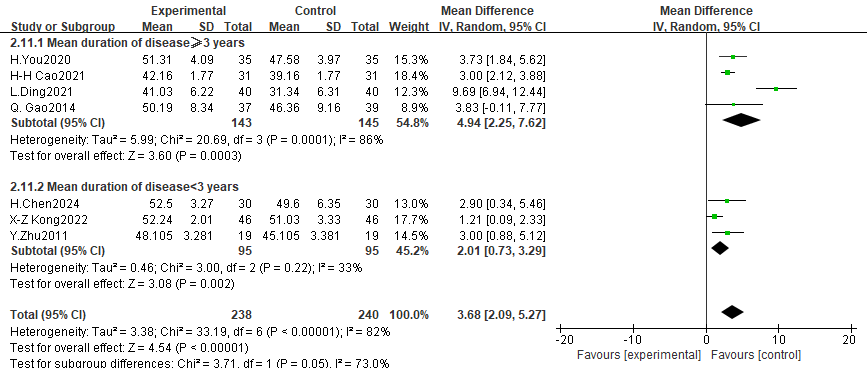


[1] Y.C. Guo, P.X. Qiu, and T.G. Liu, Tai Ji Quan: An overview of its history, health benefits, and cultural value. Journal of Sport and Health Science 3 (2014) 3-8.

[2] F.Z. Li, and P. Harmer, Economic Evaluation of a Tai Ji Quan Intervention to Reduce Falls in People With Parkinson Disease, Oregon, 2008-2011. Preventing Chronic Disease 12 (2015).

[3] P. Zeng, W. Zhang, and Z. Liu, Research and analysis of health qigong·Yijinjing of human body qi based on TCM inward observation and examination. China Journal of Traditional Chinese Medicine and Pharmacy 37 (2022) 3377-3379.

[4] Y. Kong, J. Yan, and Z. Shi, Clinical Research Progress in Health Qigong · Yijinjing. Chinese Journal of Information on Traditional Chinese Medicine 26 (2019) 133-136.

[5] K.L. Luo, X.R. Ma, X.M. Jin, X.H. Liu, Y.J. Li, S.J. Ma, and J. Hu, Effectiveness of Yijinjing on cognitive and motor functions in patients with Parkinson's disease: study protocol for a randomized controlled trial. Frontiers in Neurology 15 (2024).

[6] Y.-W. Yang, and H.-Z. Wu, Origin and development of qigong-wuqinxi. Zhonghua yi shi za zhi (Beijing, China : 1980) 41 (2011) 265-7.

[7] L. Fang, J. Yan, and K. Sun, Progress on Chinese traditional regimen of Wuqinxi exercise. China Journal of Traditional Chinese Medicine and Pharmacy 28 (2013) 837-840.

[8] T. Wang, G.P. Xiao, Z.L. Li, K.C. Jie, M.Y. Shen, Y. Jiang, Z. Wang, X.R. Shi, and J. Zhuang, Wuqinxi Exercise Improves Hand Dexterity in Patients with Parkinson's Disease. Evidence-Based Complementary and Alternative Medicine 2020 (2020).

[9] Y.T. Li, L.L. Zhang, Y. Wu, J. Zhang, and K. Liu, A Longitudinal Randomized Controlled Trial Protocol to Evaluate the Effects of Wuqinxi on Dynamic Functional Connectivity in Parkinson's Disease Patients. Frontiers in Human Neuroscience 15 (2021).

[10] J.Y. Xie, J.D. Guo, and B. Wang, Comparing the effectiveness of five traditional Chinese exercises in improving balance function in older adults: a systematic review and Bayesian network meta-analysis. Peerj 12 (2024).

[11] H.M. Yin, O.M. Cheng, X. Zhang, F.Y. Quan, Y.J. Zhang, H.Z. Zuo, J.R. Zhou, and S.Q. Xie, Effects of Liuzijue Qigong on respiratory function among patients with Parkinson's disease: a randomized clinical trial. Bmc Complementary Medicine and Therapies 25 (2025).

[12] L.Zeng, Y.Gui, J-W.Hu, H-L Yang, X-W Dong, G-L Pan, and F.Zhou, Clinical observation of acupuncture and Tuina combined with Ba Duan Jin in improving motor function in patients with Parkinson disease in the early and middle stages. Journal of Acupuncture and Tuina Science 23 (2025) 151-158.

[13] Z. Cai, and S. Dong, Effect of Ba Duan Jin Exercise on Non- Motor Symptoms, Motor Symptoms, Gait and Balance in Patients with Mild to Moderate Parkinson's Disease. Movement Disorders 33 (2018) S132-S133.
